# Feasibility of early functional rehabilitation in acute stroke survivors using the Balance-Bed—a technology that emulates microgravity

**DOI:** 10.3389/fnsys.2015.00083

**Published:** 2015-05-27

**Authors:** Lars I. E. Oddsson, Marsha J. Finkelstein, Sarah Meissner

**Affiliations:** ^1^Department of Physical Medicine and Rehabilitation, Program in Rehabilitation Science, University of MinnesotaMinneapolis, MN, USA; ^2^Technological Leadership Institute, College of Science and Engineering, University of MinnesotaMinneapolis, MN, USA; ^3^Recanati School for Community Health Professions, Ben-Gurion University of the NegevBeer Sheva, Israel; ^4^Courage Kenny Rehabilitation Institute, Allina HealthMinneapolis, MN, USA

**Keywords:** acute stroke, balance function, body weight support, countermeasure, microgravity, rehabilitation

## Abstract

Evidence-based guidelines recommend early functional rehabilitation of stroke patients when risk of patient harm can be managed. Current tools do not allow balance training under load conditions sufficiently low for acute stroke patients. This single-arm pilot study tested feasibility and safety for acute stroke survivors to use “Balance-Bed”, a technology for balance exercises in supine initially developed to emulate microgravity effects on balance. Nine acute stroke patients (50–79 years) participated in 3–10 sessions over 16–46 days as part of their rehabilitation in a hospital inpatient setting. Standard inpatient measures of outcome were monitored where lack of progress from admission to discharge might indicate possible harm. Total FIM scores at admission (median 40, range 22–53) changed to (74, 50–96), Motor FIM scores from (23, 13–32) to (50, 32–68) and Berg Balance scores from (3, 0–6) to (19, 7–43) at discharge. Changes reached Minimal Clinical Important Difference for a sufficient proportion (>0.6) of the patients to indicate no harm to the patients. In addition, therapists reported the technology was safe, provided a positive experience for the patient and fit within the rehabilitation program. They reported the device should be easier to set up and exit. We conclude acute stroke patients tolerated Balance-Bed exercises such as standing on one or two legs, squats, stepping in place as well as balance perturbations provided by the therapist. We believe this is the first time it has been demonstrated that acute stroke patients can safely perform whole body balance training including balance perturbations as part of their rehabilitation program. Future studies should include a control group and compare outcomes from best practices to interventions using the Balance-Bed. In addition, the technology is relevant for countermeasure development for spaceflight and as a test-bed of balance function under microgravity-like conditions.

## Introduction

Evidence-based clinical practice guidelines for stroke survivors strongly recommend balance training as a key component of the rehabilitation program (Ottawa et al., 2006) based on evidence that balance training improves outcomes (Sackley and Lincoln, 1997; Walker et al., 2000; Chen et al., 2002). Furthermore, there is strong evidence that higher intensity as well as more practice are important features of effective stroke rehabilitation (Van Peppen et al., 2004; Langhorne et al., 2011) especially when task-oriented training is provided early after stroke onset (Van Peppen et al., 2004). Even very early mobilization (VEM) and rehabilitation of stroke patients, within 24 h of stroke onset, has been shown to be safe and feasible in suitable patients (Bernhardt et al., 2008) and VEM may fast-track independent walking and functional recovery (Cumming et al., 2011). However, in spite of growing evidence of clear benefits to outcomes and patient’s quality of life, a recent systematic review found only three randomized controlled trials (RCTs) that initiated interventions early. Furthermore, it is commonly not part of standard clinical practice (Bernhardt et al., 2004; Veerbeek et al., 2014), although there is growing agreement that rehabilitation therapy should be provided as soon as the patient’s medical status is stable (Ottenbacher and Jannell, 1993; Cifu and Stewart, 1999). Evidence for a higher level of neuroplasticity following stroke (Murphy and Corbett, 2009) further supports early intervention as long as concerns about harm to the patient can be managed (Olavarria et al., 2014; Bernhardt et al., 2015). Consequently, it would be reasonable to assume that functional and task-oriented training early after stroke is beneficial to the patient (Veerbeek et al., 2014) and it may even be cost-effective (Tay-Teo et al., 2008). For such rehabilitation to be provided to patients early after stroke, clinicians must have access to equipment and tools that can safely deliver relevant challenges that are specific to the patient’s rehabilitation needs.

Training of gait and balance using partial body weight support (BWS; Norman et al., 1995) where the patient is strapped into an overhead harness that allows unloading of the patient’s body weight is a widely used and accepted technique in stroke rehabilitation. Improved mobility following training with BWS has been demonstrated in patients with stroke (Hesse et al., 1994, 1995; Sullivan et al., 2002; Werner et al., 2002; Barbeau and Visintin, 2003) although the concept is more commonly used in the sub-acute and chronic phases of stroke (Peurala et al., 2005; Ng et al., 2008; Ada et al., 2010; Dean et al., 2010). It has been recommended that the patient’s body weight should not be supported more than 30% (Richards et al., 1999), which is unlikely to be sufficient in the acute stage of stroke. Concerns about the balance-related fidelity of harness-based BWS training were raised by Oddsson et al. (2007) who postulated that the use of an overhead harness system constrains natural associated postural adjustments required during independent posture and gait. When supported by an overhead harness, sway of the body will be restricted by reaction forces acting through the harness that will decrease or even eliminate the need for associated postural adjustments, an important part of a natural movement repertoire. There is particular concern with limitations of mediolateral movements of the body during gait training as such displacements provide important sensory input to the brain during gait to allow for an actively controlled step-to-step mediolateral placement of the foot (Bauby and Kuo, 2000; Wall et al., 2002; Oddsson et al., 2004). In fact, differences seen in muscle activity between gait during BWS and full weight bearing training at varying velocities may reflect the decreased need for balance control and the absence of associated postural adjustments during BWS gait (Finch et al., 1991; Sullivan et al., 2002). Consequently, functional training for rehabilitation is likely to be more effective when the complexity of natural movements are incorporated (French et al., 2007) and preferably performed with slight variations between repetitions to enhance learning (Lee et al., 1991).

The Balance-Bed concept presented in Oddsson et al. (2007) was designed as a tool for rehabilitation that could provide stimulus of functional balance under low load conditions to address issues related to the lack of balance stimulus under BWS training. The system provides a supine visual environment where the subject is attached to a back pack frame and lying on a friction-free surface that allows three degrees freedom of motion in the frontal plane (mediolateral, superorinferior and roll). A single cable attached at a point on the backpack frame near the lower lumbar back of the subject provides a variable gravity-like load the subject resists during exercise. Under these circumstances the subject is mechanically unstable thus creating a natural need for associated postural adjustments to occur, and allowing challenging functional balance exercises to be performed without risk of falling. The technology has been shown to improve upright balance control as well as leg extension strength in healthy subjects (Oddsson et al., 2007).

The overall objective of this single-arm single-stage pilot study was to evaluate feasibility and safety to use the Balance-Bed for rehabilitation of balance function and mobility in acute stroke survivors undergoing inpatient rehabilitation in a hospital setting. Standard outcomes used in the clinic were monitored to indicate no harm to the participants. The study was not designed to evaluate efficacy of the system.

## Materials and Methods

This single-arm feasibility study recruited nine patients who were candidates for inpatient rehabilitation at Courage Kenny Rehabilitation Institute, Minneapolis, MN, USA. Exercises using the Balance-Bed replaced similar exercises and equipment commonly used in the rehabilitation program (e.g., the “Shuttle” by Isokinetics Inc., the patient lies supine performing leg press exercises on a bench that can slide along a track in a superoinferior direction). This study received Institutional Review Board approval from Allina Health IRB.

### Subjects

Patients who received inpatient physical therapy at the rehabilitation facility and who met inclusion/exclusion criteria were eligible for recruitment. To be included in this study participants needed to be at least aged 21 and recovering from an acute stroke and having approval/medical clearance from the treating physician to participate in the study. Participants were required to consent to participate. Women who were pregnant or individuals unable to hear, understand and follow instructions in English without the presence of an interpreter or caretaker were excluded from participation in the study. Patients having evidence or history of symptomatic orthostatic hypotension, respiratory, cardiovascular, musculoskeletal, neurological disorders or injury that prohibited participation in a moderately strenuous physical exercise program were also excluded.

### Balance-Bed Technology

**Figure 1:**
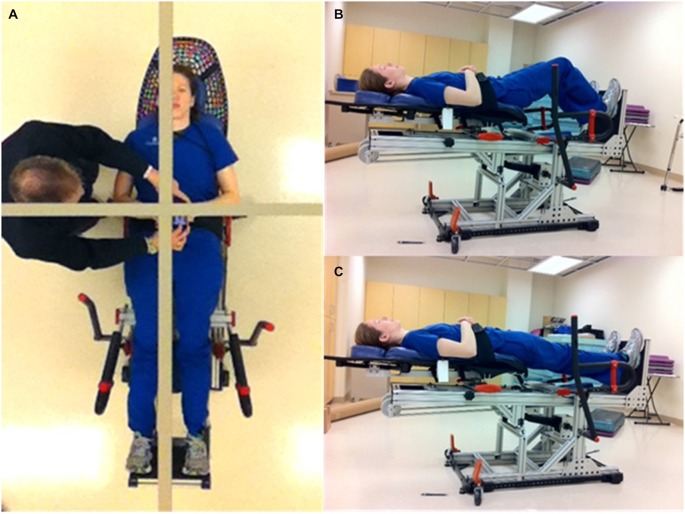
**Subject demonstrating the Balance-Bed device used in the current study**. **(A)** The ceiling mounted whole body mirror and gridline for body alignment and perceived vertical showed from subject’s perspective. **(B)** Subject in two-leg squat position. **(C)** Subject in two-leg “upright” standing position.

The concept of the Balance-Bed device system has been described in detail earlier (Oddsson et al., 2007). In brief, the device provides a tilted visual environment where the subject “stands” in a supine position while strapped to a frictionless mechanism that allows free movements in the frontal plane, similar to upright standing. A balance board or stepper can be attached to the system for more challenging activities. Sagittal plane movements cannot be performed. The embodiment of the system used in this study (Figures 1B,C) used up to six bungee cords to provide an adjustable gravity-like load (~0–60 lbs) the subject must support and balance against to remain “upright”. An assembly of low friction linear bearings mounted under a “Lazy Susan” rotary bearing provided three degrees of motion in the frontal plane for the subject. In addition, the pitch angle of the system in the current study could be increased to a near upright position to provide a gradually higher gravity-based load. Visual feedback was provided with a full body mirror mounted in the ceiling above the subject (Figure 1A). A gridline within the mirror provided a reference for the perceived vertical the subject was instructed to use to align their body to during training (Figure 1A).

### Intervention

Participants received treatment using the Balance-Bed system for at least 30 min, 1–3 times/week throughout their episode of care. The Balance-Bed treatment substituted for current standard balance/mobility interventions. Therapy progressed through a series of exercises (Table 3) that included standing, squatting, weight shifting, stepping and balance perturbations (manually provided by therapist or assistant). Subjects progressed at their own rate and the treating therapist decided what specific exercises to use, depending on the ability and state of the patient prior to each session, to provide a sufficiently challenging task. Therapists selected exercises from the Activity Log shown in Table 3 and were also able to include their own preferred exercises. Tilting the Balance-Bed added gravity-based load to performing the activity as well as a component of sensory input from the otolith tilt sensing mechanism that would be integrated into the training session.

### Standard Inpatient Outcome Measures

The standard inpatient outcome measures described below were monitored. Progress less than the established Minimal Clinically Important Difference (MCID) for each outcome was used as an indication of possible patient harm. The MCID is a measure broadly used in medical statistics as an indication of the smallest change in a clinical outcome that a patient identifies as important. The MCID Clinical outcomes were assessed at baseline and at discharge by each patient’s assigned physical therapist.

#### Functional Independence Measure (FIMTM) Total and Motor scores

The FIM was selected by a national task force sponsored by the American Congress of Rehabilitation Medicine and the American Academy of Physical Medicine and Rehabilitation to be the uniform measurement for rehabilitation outcomes related to functioning. The FIM consists of 18 items (13 motor items, 5 cognitive items). Scores on each of the items range from 0 to 7 with 7 categorized as “complete independence, score of 1 as “total assistance” and a score of 0 on the motor activities as “activity does not occur” (unsafe, medically unstable to move). Scores less than 6 indicate that another person is required for supervision or assistance (Keith et al., 1987). The motor function dimension scores range from 0 to 91. In a study of 113 patients with stroke (Beninato et al., 2006), FIM change scores associated with MCID were reported to be 22, and 17 for the FIM Total and Motor FIM Motor, respectively.

#### Berg Balance Scale (BBS)

The BBS is a 14-item performance-based instrument intended for individuals with some degree of balance impairment. Each item is scored on a 5-point scale, 0–4. Scores range from 0–56, where: <20 is confined to wheelchair, 21–45 some balance but needs assistance, 46–56 is modified independent to independent. A score of 45 or lower indicates the subject is at increased risk to fall (Berg et al., 1995). Good scores on balance scales are positively correlated with high levels of independent mobility in patients post stroke (Richards et al., 1995). The BBS MCID is defined as a difference of 5 points between measurement times if the initial score is 0–24 (Donoghue and Stokes, 2009).

### Therapist Evaluation of the Balance-Bed

The therapists who delivered the intervention were asked to complete an evaluation (Table 1) on their experience using the Balance-Bed technology. The scoring for each of the items was: 1 (Disagree strongly), 2 (Disagree), 3 (Neutral), 4 (Agree) to 5 (Strongly agree).

**Table 1:** **Therapist evaluation of the Balance-Bed**.

Statement	Agree strongly (n)	Agree (n)	Neutral (n)	Disagree (n)
Beneficial to patient	1	2	0	0
Allows safe challenge of balance	2	1	0	0
Fits within stroke rehab program	1	2	0	0
Seems to improve patients’ upright function	0	3	0	0
“Unusually” strong progress noticed	0	0	3	0
Patient appeared comfortable	0	2	1	0
Patient had positive experience	1	2	0	0
Patient was safe	3	0	0	0
Tilting is useful	1	2	0	0
Loading range is sufficient	1	1	1	0
Setup was simple	0	1	1	1
Exit was simple, (missing = 1)	0	1	0	1

### Analytical Methods

We used the Fleming Phase II one-stage model for sample size (Fleming, 1982) to determine whether the intervention had sufficient effect to warrant a future controlled study and continued development. We used the NCSS-PASS software for the estimation (NCSS LLC, Kaysville, Utah, USA). The procedure requires input of a maximum acceptable proportion (p) of subjects for a poor response and a minimum acceptable proportion of subjects for a good response. A good response was defined as improvement from baseline by at least the defined MCID in any one of the measures of function; FIM Total, FIM Motor or BBS scores, respectively. Selection of acceptable cut points for a good vs. poor response was based on our minimum tolerance for success of >50% as well as feasibility of recruitment and funding limits, which we estimated to be no more than 12 subjects during the study time period. Therefore, we selected a maximum acceptable proportion for a poor response as 0.2 and minimum acceptable proportion for a good response as 0.6. Based on these parameters, the study required 10 subjects to determine if the proportion was ≥0.6 or ≤0.2. If the number of subjects who show good responses for any of the outcomes is ≥5 the hypothesis that *p* ≤ 0.2 is rejected with a significance level of 0.03. If the number of subjects who show a good response is ≤4 the hypothesis that *p* ≥ 0.6 is rejected with a power of 83%. Outcome measures for this single-arm feasibility study were described using median and range.

## Results

### Participants

Characteristics of study participants are described in Table 2. Seven male and two females aged 50–79 received a median number of Balance-Bed sessions of six, ranging from 3 to 10 session. The low number of sessions for some patients reflected circumstances and changes in the care plan that were outside of the control of the current study, e.g., Patient #2 was discharged to a nursing home and Patient #8 participated in care giver training sessions to allow a safe discharge to home. Other circumstances included patients being discharged to outpatient rehabilitation, which made then ineligible to participate in the current study.

**Table 2:** **Subject characteristics**.

Subject	Age	Gender	LOS (days)	Time (days)	Side	Primary diagnosis	# Sessions
1	71	M	46	7	L	R MCA ischemic stroke	10
2	73	M	16	8	L + R	L cerebellar hemorrhagic stroke with craniotomy	4
3	58	M	26	5	R	L parenchymal and subarachnoid intraventricular hemorrhage	6
4	59	M	28	8	L + R	L cerebellar ischemic stroke with craniotomy	5
5	68	F	38	36	L	Hypoxic ischemic encephalopathy	9
6	75	M	37	17	L	R frontal, parenchymal and basal ganglia hemorrhagic stroke	7
7	79	M	28	9	L	R MCA ischemic stroke	5
8	79	F	29	8	L	B cerebral hemisphere and cerebellum infarcts	3
9	50	M	43	6	L	R posterior basal ganglia ischemic stroke with anaplastic astrocytoma	7
Median (Range)	71 (50–79)		29 (16–46)	8 (5–36)			6 (3–10)

The time after stroke ranged 5–36 days with a median of 8. All patients received daily rehabilitation according to recommended norms for standard of care. The median length of stay was 29 days, ranging from 16 to 46. None of the patients were able to ambulate independently at the time of their rehabilitation and had to be lifted over to the Balance-Bed from a wheel chair using a sling and a standard Hoyer hydraulic lift to conduct the training session. None of the patients reported nausea or dizziness during the training sessions. Patient #3 requested to cancel one training session due to discomfort in his right great toe that may have been aggravated during a previous session on the Balance-bed. Patient #9 experienced a disruption in rehabilitation treatment between sessions 4 and 5 to receive treatment for deep vein thrombosis and pulmonary embolism, unrelated to the Balance-Bed training.

### Balance-Bed Activities

The different training exercises used for the Balance-Bed sessions are shown in Table 3. The treating therapist selected exercises for each session that would best target the specific need for each patient. Table 3 shows which exercises each patient experienced on the Balance-Bed throughout their episode of care. Each number, 1 through 9 in the “Patient #” column, represents a patient undergoing that specific exercise during one session. Since a majority of the subjects had their left side affected, the most commonly selected exercise was squat performed on the left leg (Table 3). Details of each subject’s activity log will not be reported here. As examples of overall activity, Patient #1 performed between 40–250 repetitions/session for a total of 1490 repetitions across the whole episode of care (46 days, 10 Balance-bed sessions). Patient #5 performed between 40–100 repetitions/session in the Balance-Bed for a total of 625 repetitions across the episode of care (38 days, 9 Balance-Bed session). Exercises typically included one and two-leg squats with eyes open or closed (Table 3). Standing exercises typically lasted 2–25 min and were commonly conducted to help the patient find “their midline” based on visual feedback from the body mirror and were also combined with bouts of balance perturbations. Therapists used the tilt feature of the Balance-Bed to gradually increase the overall load of an exercise. The largest amount of tilt used in the current study was about 40°, which corresponded to 64% of full body gravity-based load. A 10 degrees tilt was more commonly used, corresponding to an added 17% of full body gravity-based load.

**Table 3:** **Activity Log used for Balance-Bed training sessions**.

Exercise/Activity	Patient #
Standing 2-leg normal base EO	112222333344445566666777
Standing 2-leg narrow base EO	3
Standing 2-leg normal base EC	3444
Standing 2-leg narrow base EC	
Standing R-leg EO	449
Standing L-leg EO	11111111449
Standing R-leg EC	4
Standing L-leg EC	14
Standing weight shift EO	222248889999999
Standing weight shift EC	99
Squat 2-leg EO	11222233333444445555556666666777778889999999
Squat 2-leg EC	3333444456
Squat R-leg EO	233344444566667
Squat R-leg EC	34
Squat L-leg EO	1111111111233344444555555555666666677777999999
Squat L-leg EC	34
Step-in-place EO	2224668
Step-in-place EC	
Perturbations	11112222344444556666677788899

*Acronyms: EO, Eyes open; EC, Eyes closed; R, Right; L, Left. A separate log was used for each patient and each session. Column with Patient # indicates which exercise patients experienced and for how many session, e.g., 449 for the exercise “Standing R-leg EO” means that patient #4 experienced this exercise at two sessions and patient #9 at one session. The following information (not shown in the Table) was also collected for each training session: Duration (min), # Reps, # Sets, Foam (Y/N), Tilt (deg), Load (#cords)*.

All patients were exposed to manually delivered balance perturbations, provided either as a brief lateral push at the hip or shoulder level or as a sudden release of a lateral “hold” the subject resisted while maintaining a stable position. Balance perturbations were typically delivered in bouts of 5–15 and repeated two to three times depending on subjects’ ability and level of fatigue. Although not measured objectively, the ability of patients to recover from balance perturbations appeared to improve over time. The following comments in the Activity Log for Patient #6 by the treating therapist illustrate from a subjective perspective an enhanced ability throughout the episode of care to better react to balance perturbations during supine “standing”:

- Session 1: *“Attempted to have patient resist perturbations with both lower extremities support with minimal success, patient able to resist some, but unable to correct once out of midline even with cues. Patient struggles to use mirror for visual feedback.”*
- Session 3: *“He was able to withstand some perturbations both directions. Patient needing continuous cues throughout the session to look up at the mirror instead of his R gaze preference.”*
- Session 5: *“Worked on maintaining midline and balance with mirror for visual feedback and bilateral knee extension while staff provided perturbations. Able to correct mild perturbations towards left.”*
- Session 6: *“Worked on maintaining midline and balance with mirror for visual feedback and bilateral knee extension while staff provided perturbations. Able to correct max perturbations towards left.”*
- Session 7: *“Able to maintain midline and balance with mirror for visual feedback and bilateral knee extension while staff provided perturbations. Able to correct max perturbations towards left side without cues for left knee.”*

### Standard Inpatient Outcome Measures

Participant outcomes that were used to indicate no harm are shown in Table 4. Seven, eight and seven of the first nine participants achieved MCID on FIM Total, FIM Motor and BBS scores, respectively. Consequently, since the proportion of patients required to indicate progress (“good response”) exceeded 0.6 (7/9 = 0.78) after nine patients had been treated it was decided to close the study (if a 10th patient would have shown poor response in outcomes the proportion would be 7/10, which is still larger than the required 0.6). Patient #2, the only subject not to achieve MCID in at least one of the three measures, was discharged early to a nursing home.

**Table 4:** **Standard inpatient outcome measures for participating patients showing values at admission (Pre), discharge (Post) as well as change for total Functional Independence Measures (FIM), FIM Motor and Berg Balance Scale (BBS) scores**.

Subject	FIM total pre	FIM total post	FIM total change	FIM motor pre	FIM motor post	FIM motor change	BBS pre	BBS post	BBS change	# Sessions
1	22	72	50*	13	44	31*	3	11	8*	10
2	37	50	13	24	32	8	6	7	1	4
3	47	95	48*	32	68	36*	4	43	39*	6
4	43	96	53*	28	65	37*	4	22	18*	5
5	39	60	21*	17	37	20*	0	N/A	N/A	9
6	40	66	26*	18	43	25*	2	15	13*	7
7	39	82	43*	17	55	38*	1	33	32*	5
8	53	87	34*	25	59	34*	4	23	19*	3
9	43	74	31*	23	50	27*	1	16	15*	7
Median	40	74	34	23	50	31	3	19	16.5	6
Range	22–53	50–96	13–53	13–32	32–68	8–38	0–6	7–43	1–39	3–10

*Measures were used to demonstrate no harm to the patients if changes exceeded MCID for each of the outcomes (22, 17, and 5 for FIM Total, FIM Motor, and BBS, respectively). Patients who reached MCID for each of the outcomes are indicated with an asterisk*.

### Therapist Evaluation

Table 1 shows results from the therapist evaluation. Three of five treating therapists completed the evaluation. There was general agreement that the intervention was beneficial to the patients and allowed a safe way to challenge balance, that the tilting feature was useful, the intervention fit within the stroke rehabilitation program, and provided a positive experience for the patient. All therapists were “neutral” to the statement about strong progress. Mixed responses were seen regarding sufficiency of the loading range and simplicity of the setup. There were no “strongly disagree” responses to any of the questions (Table 1).

## Discussion

This single-arm study has demonstrated the feasibility and safety of an intervention that provides whole body functional balance exercises in a supine position for acute stroke patients. A higher than the minimum proportion of patients required for a good response (0.6), as indicated by a change in outcome of at least the clinically accepted MCID, was achieved for seven, eight and seven of the nine participating patients on FIM Total, FIM Motor and BBS scores, respectively. It is important to note that changes seen in outcomes cannot be attributed to the use of the Balance-Bed. This study was not designed to compare changes in outcomes with a control group and patients received additional rehabilitation during their episode of care where the Balance-Bed was not used. Instead, the findings suggest the device is feasible, does not harm the patients and that further study of the technology in a controlled intervention study would be merited. Changes in outcome scores were in line with historical data at the rehabilitation clinic and similar to those reported in the literature for patients similar to our participants (Inouye et al., 2001), which lends further support to the conclusion that the intervention did no harm to the patients. Furthermore, treating therapists agreed that the intervention was safe and beneficial to the patients and fit within the rehabilitation program.

The Balance-Bed environment allowed whole body functional balance exercises to be performed at levels of load as low as ~10% of body weight up to loads above full body weight (when system is tilted to upright), where the lower load levels can be fully sustained by acute stroke patients. Richards et al. (1999) recommended that the body weight of patients using BWS systems should not be supported more than 30%. At higher support levels patients would more or less hang in the harness, which is unlikely to be comfortable nor helpful for patients in the acute phase of stroke. Furthermore, BWS systems alter the need for natural associated postural adjustments during standing and walking exercises, which may later jeopardize stability and control of independent gait (Oddsson et al., 2007). These systems may prevent falls during rehabilitation but they also prevent the development of relevant reactive postural adjustments. On the other hand, as demonstrated in the current study, mediolateral balance perturbations can be safely trained in the Balance-Bed environment where a “fall” can occur with no risk of injury. All nine of the participating patients were exposed to balance perturbation training. We believe this is the first time it has been shown that acute stroke patients can be safely exposed to balance perturbations without the use of a safety harness that prevents the initiation and development of functional postural recovery strategies. This advances our understanding of the kind of exercises acute stroke survivors are able to perform when the right tools are available in the clinic.

Based on input from the therapists, some features of the technology as used in this study need to be improved. This includes how to enter and exit the system and incorporating a limb support for use during single leg stance. Visual feedback for the training environment was provided from a whole body mirror mounted in the ceiling above the subject (Figure 1A). This appeared to be helpful and sufficient for the subject to use as help during balance training to align the body with a gridline in the mirror as a representation of their perception of upright. Previous embodiments of the system, however, used more advanced technologies to achieve a more fully immersed experience for the subject including a small tilted room with familiar objects surrounding the subject as well as large glasses-free automultiscopic 3D screens placed around the subject for a virtual reality experience (Oddsson et al., 2007). More recently developed virtual reality systems, such as the Oculus Rift, may provide an additional way to provide an immersive experience with the Balance-Bed system.

The Balance-Bed technology is also relevant for space flight as a ground-based emulation of microgravity effects on the balance control system. In a supine position on earth or any other gravity field, the otolith tilt sensing mechanism in the vestibular system cannot contribute to balance control leaving the subject to rely mainly on sensory information from the visual and somatosensory systems for balance control, similar to what would be the case in a microgravity environment (Oddsson et al., 2007). It is well known that astronauts lose postural control and coordination during exposure to microgravity, especially following long-term flights (Koppelmans et al., 2013). These problems may influence safety during re-entry into the atmosphere and egress of the vehicle after landing. Furthermore, astronauts have prolonged post flight balance-related problems during common everyday activities such as standing, walking and turning corners. Previous results using the Balance-Bed technology suggest that astronauts training in-flight under microgravity conditions should be able to conduct exercises that stimulate postural control as well as muscle strength in parallel. In addition, this technology may be used to develop outcomes that reflect the function of the balance control system under circumstances when otolith tilt information is absent or unreliable. It could also be used as a training environment to improve reliance on visual and somatosensory cues for balance control preparing astronauts for extended periods of spaceflight required for interplanetary travel.

## Conclusion

The Balance-Bed technology is a novel tool to help therapists provide whole body functional balance exercises, including balance perturbations, for patients in the acute phase of stroke. Additional categories of patients in need of functional balance exercises before they are able to fully support themselves upright against gravity may also benefit from its use. Our findings from this feasibility study justify future studies designed to specifically compare outcomes from best practices for rehabilitation to interventions using the Balance-Bed. Such studies should incorporate outcomes that may be more specific for balance function such as the Postural Assessment Scale for Stroke Patients. Future studies should target several areas including patients in an outpatient setting. In the current study, some patients were discharged early from inpatient rehabilitation and referred to outpatient therapy, which made them ineligible for continued participation. In addition, the Balance-Bed technology may be useful for patients as a tool for very early mobilization, i.e., within 24 h of their stroke. We hypothesize that balance related outcomes will be improved compared to current expectations since currently available tools cannot provide a realistic challenge to the balance control system.

## Conflict of Interest Statement

Dr. Oddsson is inventor of the technology used in the current study. He is the owner of two issued patents on this technology. The technology has not been commercialized and Dr. Oddsson is not receiving any royalties or licensing fees. The other authors declare that the research was conducted in the absence of any commercial or financial relationships that could be construed as a potential conflict of interest.
